# Everolimus-based immunosuppression induces alloreactive regulatory T cell expansion combined with loss of alloreactive effector T cells

**DOI:** 10.3389/fimmu.2026.1904559

**Published:** 2026-08-03

**Authors:** Nicolle H. R. Litjens, Jip Jonker, Mariska Klepper, Frederique Prevoo, Dennis A. Hesselink, Frederike J. Bemelman, S. Azam Nurmohamed, Arjan D. Van Zuilen, Stefan P. Berger, Jan-Stephan F. Sanders, Michiel G. H. Betjes

**Affiliations:** 1 Erasmus Medical Center (MC) Transplant Institute, Department of Internal Medicine, Division of Nephrology and Transplantation, University Medical Center Rotterdam, Rotterdam, Netherlands; 2 Department of Internal Medicine, Division of Nephrology, University Medical Center, Groningen, Netherlands; 3 Department of Nephrology, Amsterdam University Medical Center, Amsterdam, Netherlands; 4 Department of Nephrology, University Medical Center Utrecht, Utrecht, Netherlands

**Keywords:** human, immunosuppressive regimens, T cells, tolerance/suppression/anergy, transplantation

## Abstract

**Background:**

Mammalian target of rapamycin inhibitors (mTORi) combined with tacrolimus and prednisolone enable tacrolimus dose reduction. This study evaluated everolimus–low-dose tacrolimus–prednisolone (EVR-IS) versus standard myco-phenolate mofetil (MMF)–tacrolimus–prednisolone (MMF-IS) on effector (Teff) and regulatory T cells (Tregs) in elderly kidney transplant recipients.

**Methods:**

Blood samples were obtained before, and 12 and 24 months after transplantation in the OPTIMIZE study (EudraCT 2018-003194-10). Recipients ≥ 65 years were randomized to EVR-IS or MMF-IS. Phenotype of circulating T cells (EVR-IS N = 86; MMF-IS N = 83) was assessed by multiparameter flow cytometry including transcription factors. Exploratory immune profiling in a limited cohort of kidney transplant recipients, involved detailed characterization of function and phenotype of donor antigen-specific alloreactive and virus-specific T cells, identified by activation-induced CD137 expression using multiparameter flow cytometry. Treg function was analyzed using a suppression assay after isolation and expansion of Tregs.

**Results:**

Effector memory T cells remained stable under EVR-IS but showed a small decline for MMF-IS. Treg frequencies increased in EVR-IS recipients (2.9% to 4.1%, P<0.01). Under EVR-IS, donor antigen-specific alloreactive CD4+ T cells remained stable whereas they declined under MMF-IS. Furthermore, donor antigen-specific alloreactive CD8+ T cells declined more rapidly under EVR-IS than for MMF-IS. Only under EVR-IS donor antigen-specific alloreactive Tregs (3.9% to 5.8%, P = 0.03) increased, which strongly suppressed donor antigen-specific alloreactive effector T-cell proliferation. This led to significantly higher Treg/CD4+Teff (3-fold, P<0.01) and Treg/CD8+Teff (10-fold, P = 0.01) ratios for EVR-IS at M24, respectively. Both regimens reduced polyfunctional donor antigen-specific alloreactive CD4+ T cells during the first year, while proportions of virus-specific T-cells remained unchanged.

**Conclusion:**

This study demonstrated that everolimus-combined with low dose tacrolimus and mycophenolate mofetil combined with standard dose of tacrolimus employ different mechanisms to effectively control the alloimmune response facilitating patient-tailored immunosuppression in elderly kidney transplant recipients.

## 1 Introduction

The clinical introduction of calcineurin inhibitors (CNI), in particular tacrolimus, has reduced the rate of acute rejection after kidney transplantation and has increased the 1-year survival rate (1). However, the major concern with long term use of CNI is its nephrotoxicity alongside the increased probability to develop post-transplant diabetes mellitus and hypertension (2). Mammalian target of rapamycin inhibitors (mTORi) in combination with low dose CNI are considered a CNI-sparing alternative to the combination with mycophenolate mofetil (3).

The next generation mTOR inhibitor everolimus, a rapamycin-analog, has been used successfully after kidney transplantation in combination with reduced-exposure CNI (tacrolimus and cyclosporine) and prednisolone. The incidence of acute rejection and graft survival and function was similar compared to standard dose of CNI combined with MMF and prednisolone, but the everolimus group showed a decreased incidence of viral infections (3). Recipients receiving a kidney from an older donor are at increased risk of CNI-induced nephrotoxicity (4). Therefore, less exposure to CNI may be particularly beneficial in the Eurotransplant Senior Program, where older donor kidneys are preferentially allocated to older (≥ 65 years) recipients. Within the OPTIMIZE trial this hypothesis was tested by comparing a treatment arm with reduced-exposure tacrolimus combined with everolimus and prednisolone versus standard-exposure tacrolimus (tacrolimus/MMF/prednisolone) (5). The recently published results showed no difference in kidney transplant function at 12 and 24 months, a similar low rate of rejection and no difference between the treatment arms for incidence of viral infections (6). Everolimus inhibits mTOR which is a member of the family of phosphatidylinositol-3 kinase-related kinases. Inhibition leads, amongst others, to less T cell proliferation and diminished cellular responses to several cytokines (7). However, the impact of everolimus on circulating T cells and especially on alloreactive T cells is largely unknown. An interesting feature of mTORi, in the context of its use as an immunosuppressant, is its potential to promote the expansion of regulatory T cells *in vitro* (8). Such an expansion of regulatory T cells after solid organ transplantation may contribute to graft acceptance. However, conflicting reports have been published on Treg expansion in humans using rapamycin and, importantly, it is not known whether donor antigen-specific alloreactive Tregs are specifically expanded.

The introduction of activation-induced markers like CD137 and CD154 enabled the identification of antigen-reactive effector and regulatory T cells at the single cell level with simultaneous multiparameter analysis of their differentiation state and function (9–12). Recent findings using these markers showed that activation-induced cell death of polyfunctional donor-specific alloreactive CD4+ T cells is associated with donor-specific hypo-responsiveness after kidney transplantation (13, 14). In this study we analyzed the changes of T cell subsets and the dynamics of donor antigen-specific alloreactive effector and natural regulatory T cells in recipients receiving an immunosuppressive regimen with either reduced-exposure tacrolimus and everolimus (EVR-IS) or standard-exposure tacrolimus and MMF (MMF-IS).

## 2 Materials and methods

### 2.1 Study population

Peripheral blood was obtained from kidney transplant recipients participating in the OPTIMIZE trial (EudraCT number: 2018-003194-10) at time of transplantation, and 12 months and 24 months after transplantation. All patients gave written informed consent to participate in this trial and to donate peripheral blood for T cell analysis (METC 2018.698). Peripheral blood mononuclear cells (PBMCs) from living donors were collected as part of the biobank at the Erasmus Medical Center (METC 2012-022) for which written informed consent was obtained. Within the OPTIMIZE trial, recipients of ≥ 65 years were included and randomized to either an everolimus- or MMF-based immunosuppressive regimen (5). After 3 months, either a regimen with low-dose tacrolimus (trough levels 1.5-4 μg/L), everolimus (trough level 3-6 μg/L) and prednisolone 5 mg was given or a maintenance immunosuppressive regimen of standard-dose tacrolimus (trough level 5-8 μg/L), MMF 500 mg bid and prednisolone 5 mg. All recipients received induction therapy with the anti-CD25 antibody basiliximab. Patients were excluded if they presented with very low (HLA-identical living donor) or high immunologic risk, received an ABO-incompatible allograft, or had a positive CDC cross-match. Additional exclusion criteria included signs of active infection or a high probability of severe, unacceptable side effects from the study medication. This study was conducted in accordance with the Declaration of Helsinki and the Declaration of Istanbul and in compliance with International Conference on Harmonization/Good Clinical Practice regulations.

### 2.2 PBMCs isolation

PBMCs were isolated from heparinized peripheral blood as described previously (15) in a selection of the study centers as not all centers withdrew blood for research purposes from the kidney transplant recipients included in the trial. Moreover, in some centers also donor material, i.e. being PBMCs or spleen mononuclear cells were collected, facilitating studies into donor-specific alloreactive T cell responses. PBMCs and spleen mononuclear cells were stored at 10x10^6^/vial until further use. Analyses of samples were performed in batches, pooling the different time points of a patient in one assay to minimize inter-assay variation.

### 2.3 T cell phenotype analysis

Absolute numbers of T cells were determined by whole blood staining and analysis by flowcytometry as described before (16). Multiparameter flowcytometry was used to characterize circulating T cells in more detail. For this purpose, PBMCs were thawed and following staining of dead cells, using the fixable viability stain (FVS)-780 (BD Biosciences Erembodegem, Belgium), cells were stained at the cell surface using antibodies to allow for characterization of the different T cell subsets followed by intracellular staining upon permeabilization (**Supplementary Table 1**) using the eBioscience™ FOXP3/Transcription Factor Staining Buffer set according to manufacturer’s instructions (Thermo Fisher Scientific, Bleiswijk, Netherlands) to be able to analyze expression of transcription factors. Samples were measured on the Symphony A3 light (BD Biosciences). We stored 0.5–1 million viable T cells during measurement for subsequent characterization of T cell subsets using Kaluza version 2.1 software (Beckman Coulter, Woerden, Netherlands). A representative example of the gating strategy used for analysis of the different CD4+ T cell subsets is depicted in **Supplementary Figure 1** and a similar strategy was employed for CD8+ T cell subsets. In addition, unbiased analysis of data was performed using the online platform Omiq. Using the flowSOM algorithm (17), cells were clustered based on similar expression profiles by considering the median fluorescence intensity of all markers on a per cell basis including the whole spectrum of expression, ranging from high to low expression.

### 2.4 Identification of polyfunctional donor antigen-specific alloreactive T cells and virus-peptide reactive T cells

In sub studies, we analyzed function and phenotype (**Supplementary Tables 2A–C**) of antigen-reactive T cells. For these sub studies, we could only include recipients that had sufficient cells isolated, a complete follow-up, remained on the same IS regimen throughout follow-up period and for which also donor material was collected. Antigen-reactive T cells were identified by expression of the activation-induced CD137 upon a short-term stimulation of PBMCs with antigen (10). Frequencies of cytokine producing T cells within CD137-expressing antigen-reactive T cells were assessed upon stimulating recipient PBMCs for 15 hours with CD3-depleted donor cells (ratio 1:0.5) in presence of αCD49d (1 μg/mL, BD Biosciences), a protein transport inhibitor cocktail (eBioscience™/Thermo Fisher Scientific) and FITC-labelled anti-CD107a (Biolegend). As a negative control, cells were left unstimulated. In addition, a mixture of overlapping peptide pools (Miltenyi Biotec) for cytomegalovirus (CMV pp65 and IE-1) and Epstein-Barr virus (EBV consensus) were used to evaluate virus-related immune responses for both treatment regimens. For this, cells were preincubated with this mixture for two hours, to allow uptake and presentation of these antigens, before adding the protein transport inhibitor cocktail. These peptide pools consist of 15-mer peptides with 11 amino acid overlap allowing for characterization of both reactive CD4+ as well as CD8+ T cells within PBMCs.

Upon stimulation, cells were stained at the cell surface and intracellular as described before (14) using antibodies listed in **Supplementary Table 3**. Samples were measured on the Symphony A3 light (BD Biosciences). Between 0.5–1 million viable T cells were stored during measurement for subsequent analysis using Kaluza version 2.1 software (Beckman Coulter). Polyfunctional T cells were defined as cells expressing at least 2 cytokines after antigen stimulation (13). A representative example of gating strategy for analysis of polyfunctional CD4+ T cells is depicted in **Supplementary Figure 2** and a similar approach was employed for CD8+ T cells.

### 2.5 Identification of donor antigen-specific alloreactive regulatory T cells

Natural occurring CD4+ regulatory T cells (Tregs) are identified as CD4+ T cells highly expressing CD25 (alpha-chain of the IL-2 receptor) and lacking CD127 (IL-7 receptor). As previously described, within this cell population, the Treg-specific demethylated region (TSDR) within the *FOXP3* promotor is highly demethylated and it harbors highly immunosuppressive cells with antigen reactivity for several antigens, including donor antigen-specific alloreactive Tregs (12). Short term culture blurs the differentiation of Tregs and effector T cells based on CD25/CD127 expression. Therefore, PBMCs of kidney transplant recipients were thawed, allowed to rest for 6 hours and stained prior to stimulation with antibodies directed against CD25 (BV711-labelled CD25, clone Biolegend Europe BV, Amsterdam, Netherlands) and CD127 (PE-Cy7-labelled CD127, Biolegend), ensuring identification of CD4+ Tregs upon short term culture (**Supplementary Figure 3**). Stimulation with or without donor cells (at a ratio of 1:0.5) for 18–24 hours (13, 14) was performed in the presence of anti-CD49d (1 μg/mL, BD Biosciences) to facilitate characterization of T cells with a higher activation threshold. Proportions of donor antigen-specific alloreactive Tregs, CD4+ and CD8+ T cell subsets (10) were measured by flow cytometry upon staining the cells at the cell surface (**Supplementary Table 4**). A representative example of gating strategy for analysis of CD137-expression within T cells is depicted in **Supplementary Figure 4**).

### 2.6 Isolation and expansion of Tregs

Suppressive capacity of Tregs was analyzed in 10 kidney transplant recipients of which 5 were on EVR-IS and 5 on MMF-IS (**Supplementary Table 2D**). Regulatory T cells were sorted using flowcytometry-based cell sorting (FACS Aria II SORP, BD Biosciences) as previously described (12, 18). For these experiments, we included M12 or M24 samples from 5 kidney transplant recipients on EVR-IS and 5 on MMF-IS. Purity of Tregs was >95%. The yield of Tregs was too low to evaluate suppressive function immediately following sorting. Therefore, Tregs were expanded in a polyclonal manner using αCD3/αCD28-coated beads (Dynabeads^®^ Human T-Activator CD3/CD28 beads, Thermo Fisher Scientific) at a ratio 1 bead:1 cell as described before (18) and when sufficient Tregs were obtained following sorting, part of the Tregs were also expanded in an antigen-specific manner using irradiated (30 Gy) donor cells at a ratio of 4 PBMCs:1 Treg. Expansion was done for 9–11 days in the presence of recombinant human IL-15 (10 ng/mL; Thermo Fisher Scientific) and IL-2 (25 U/mL; Proleukin^®^, Clinigen Healthcare Ltd, Burton, UK).

### 2.7 Suppression assay

Following expansion, Tregs were harvested and tested for suppressive capacity in a mixed lymphocyte reaction as described previously (18) using CFSE-dilution as a read-out instead of [^3^H] thymidine incorporation. Briefly, donor-reactive and polyclonal expanded Tregs were labeled with PKH-26 (Merck Life Science NV, Amsterdam, Netherlands) according to manufacturer’s instructions to prevent convergence of proliferated CFSE- and PKH-26-negative cells. These Tregs were then added in triplicate in a 96-well plate at different ratios ranging from 1:4-1:256. Subsequently, PBMCs of kidney transplant recipients prior to transplantation, used as responder cells, were labelled with CFSE (Thermo Fisher Scientific) according to manufacturer’s instruction. The CFSE-labeled PBMCs were added in triplicate to the wells at 5x10^4^ per well and stimulated with irradiated donor cells. No stimulation and stimulation with αCD3/αCD28-coated beads were included as negative and positive control (ratio beads:cells 1:1), respectively. Following stimulation for 6 days, effects of Tregs were evaluated by analyzing CFSE-dilution using flowcytometry. Samples were measured on the Symphony A3 light (BD Biosciences). We aimed for acquiring 50.000 viable T cells for subsequent analysis of proliferation using Kaluza version 2.1 software (Beckman Coulter). Inhibition of proliferation was determined by calculating the difference between proliferating T cells in absence of Tregs to that in presence of Tregs and relating this difference to the proliferation in absence of Tregs.

### 2.8 Statistical analyses

Statistical analyses and graphs were created with GraphPad Prism 9.0.0 (GraphPad Software Inc., San Diego, CA, USA). Normally distributed data are depicted as mean and standard error of the mean (SEM), non-normally distributed data as median and interquartile range (IQR). The two-way ANOVA model cannot be used due to missing values and we therefore used the mixed effect model to compare the effect of time (listed in rows) and/or treatment arms (listed in columns) for the different read-outs. *Post-hoc* analyses correcting for multiple testing (Tukey/Šídák multiple comparison test) were used to evaluate where significant effects were observed in time or between the two treatment arms. A P-value<0.05 is considered statistical significant.

## 3 Results

### 3.1 Study population characteristics

The study population characteristics are depicted in **Table 1**. One hundred sixty nine participants in the OPTIMIZE trial were included for this study of which 86 were randomized to EVR-IS and 83 to MMF-IS. No significant differences were observed between the two treatment arms with respect to baseline demographic characteristics and rejection incidence, mortality and infections during two-year follow up (**Table 1**).

**Table 1:** Study population characteristics.

Characteristic	EVR-IS	MMF-IS	P-value
Number of individuals	86	83	
Recipient age in years, median [IQR]*	70 (67-74)	70 (67-74)	0.88
Recipient Male, n(%)	62 (72%)	60 (72%)	1.00
Donor age in years, median [IQR] *	69 (64-71)	68 (62-72)	0.89
Donor Male, n(%)	42 (49%)	41 (49%)	1.00
DSA, n(%)*/**	6 (7.5%)	8 (9.9%)	0.78
First kidney transplant, n(%)	84 (98%)	79 (95%)	0.44
Type of transplantation			0.88
Living donor, n(%)	32 (37%)	29 (35%)	
Deceased donor, n(%)	54 (63%)	54 (65%)	
Number of mismatches HLA, median [IQR]			
HLA class I	3 (2-3)	3 (2-3)	0.98
HLA class II	1 (1-2)	1 (1-2)	0.73
Renal replacement therapy, n(%)			0.65
No (pre-emptive)	29 (34%)	25 (30%)	
Dialysis	57 (66%)	58 (70%)	
CMV serostatus acceptor, n (%)*			0.48
Seropositive	44 (51%)	47 (57%)	
Seronegative	42 (49%)	36 (43%)	
Biopsy proven acute rejection, n(%)***	10 (12%)	9 (11%)	>0.99
CMV infection, n(%)***	8 (9%)	9 (11)	0.80
Infection total (bacterial and viral), n(%)***	64 (74%)	69 (83%)	>0.99
Mortality, n(%) ***	6 (7%)	10 (12%)	0.30

* at time of transplantation.

** number of individuals for which DSA were determined different from total included for this study.

*** occurring after transplantation.

### 3.2 Circulating T cell subsets after kidney transplantation

In both treatment arms the number of CD3+ T cells remained similar up to 24 months after transplantation without a change in CD4+/CD8+ ratio (**Supplementary Figures 5A–C**). The average CD3+ T cell numbers amounted to 1.1x10^3^ cells/μL and 1.4x10^3^ cells/μL versus 1.2x10^3^ cells/μL and 1.3x10^3^ cells/μL prior to and M24 after transplantation for EVR- and MMF-IS, respectively.

**Figure 1:**
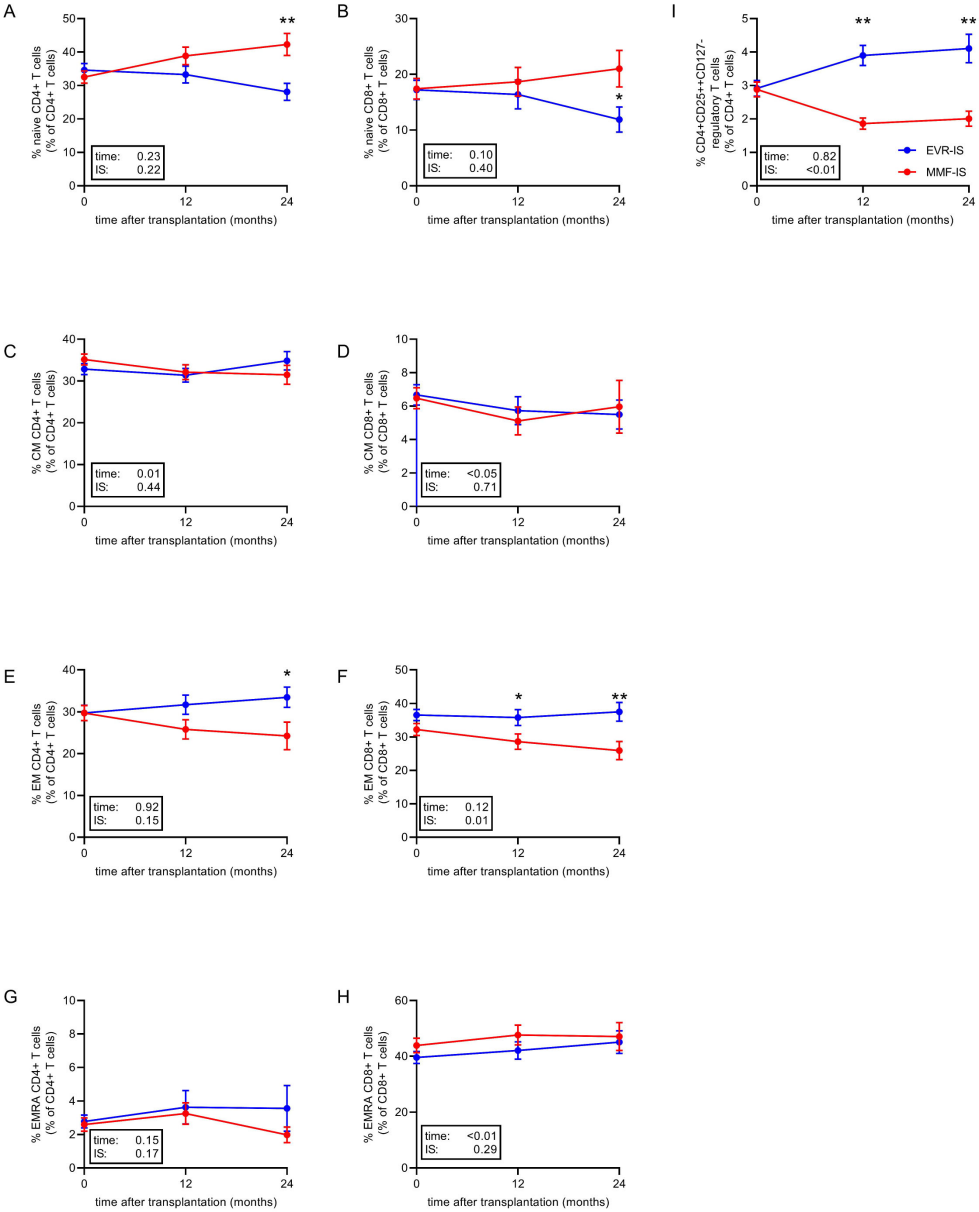
Composition of circulating CD4+ and CD8+ T cell subsets. Circulating CD4+ and CD8+ T cell subsets were determined by multiparameter flowcytometry for 169 kidney transplant recipients prior to and at 12 and 24 months after transplantation. On the Y-axis, percentages for each subset are depicted whereas on the X-axis, time after transplantation is given. Naive **(A, B)**, CM **(C, D)**, EM **(E, F)**, EMRA **(G, H)** CD4+ and CD8+ T cells, respectively are depicted. CD4+CD25++CD127- T cells (Tregs) are given in **(I)**. The red lines reflect mean ± SEM values for recipients on MMF-IS (N = 83) whereas the blue lines reflect those for recipients on EVR-IS (N = 86). Mixed effect analysis with multiple comparison test was used to determine effect of time and treatment arm (IS) on the kinetics of T cell subsets (P-values are listed in square) and to compare the difference between both treatment arms for each time-point. * represents a P-value <0.05 and ** a P value <0.01.

MMF-IS resulted in a significant decrease of percentages of central memory (Tcm, **Figure 1C**), effector memory (Tem, **Figure 1E**) CD4+ as well as CD8+ Tem (**Figure 1F**) cells at M12. Proportions of CD4+ Tcm and Tem decreased from 35.2 ± 1.3% and 29.7 ± 1.8% prior to transplantation to 32.1 ± 1.8% and 25.8 ± 2.3% at M12 for Tcm and Tem, respectively. In addition, proportions of CD8+ Tem decreased from 32.2 ± 2.3% prior to transplantation to 28.6 ± 2.3% at M12, respectively. This decrease in memory T cell subsets was accompanied by opposite relative changes in naïve T cells (**Figures 1A, B**). In the EVR-IS group percentages of CD4+ (**Figure 1E**) and CD8+ (**Figure 1F**) Tcm and Tem remained similar after transplantation leading to a significant difference at M24 in the percentages of CD4+ Tem (P = 0.03) and CD8+ Tem cells (P<0.01) between both IS regimens.

EVR-IS led to an increased percentage of Tregs at M12 (from 2.9 ± 0.2% prior to transplantation to 3.9 ± 0.3% at M12 after transplantation, P<0.01, **Figure 1I**) and 4.1 ± 0.4% at M24. In contrast, the percentage of CD4+ Tregs in the MMF-IS arm decreased in time from 2.9 ± 0.2% prior to transplantation to 1.9 ± 0.2% at M12 (P<0.01, **Figure 1I**) and 2.0 ± 0.2% at M24.

Overall, the analysis of T cell subsets by classical definitions revealed two major changes between the EVR-IS and MMF-IS groups: The EVR-IS group showed a significant expansion of Tregs while effector memory T cells remained stable whereas for the MMF-IS group a significant decrease in both Tregs and effector memory T cells was observed.

### 3.3 Unbiased analysis of differences in T cell subsets between treatment arms in time

**Figure 2:**
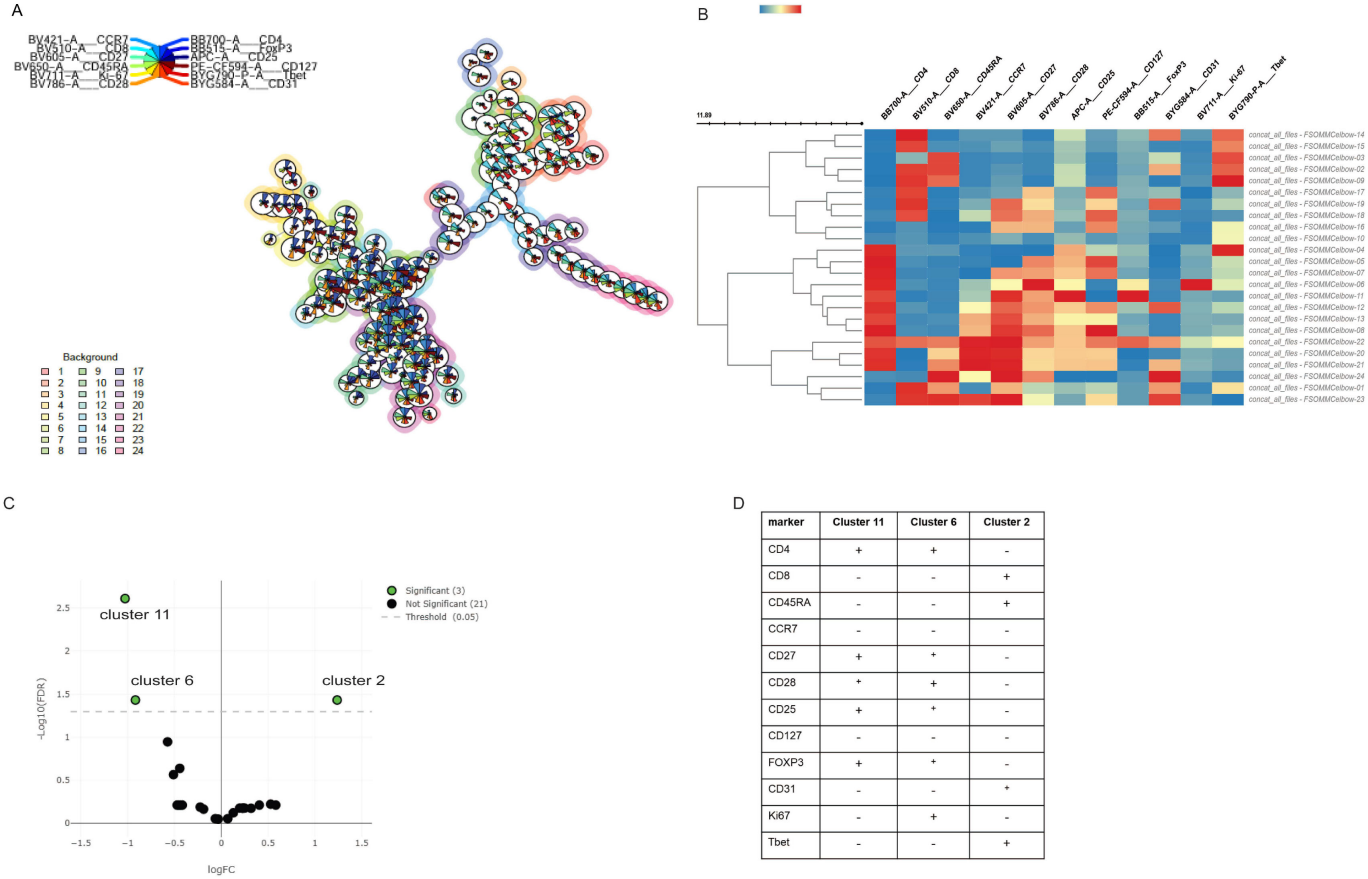
FlowSOM of circulating CD3+ T cells. Circulating T cells were characterized using multiparameter flowcytometry for kidney transplant recipients receiving either MMF-based IS or EVR-based IS prior to and at 12 and 24 months after transplantation. All these measurements were analyzed in an unbiased manner using FlowSOM. In **(A)**, the resulting minimal spanning tree is depicted with 24 different clusters being identified. Each node depicts the expression of all different markers as parts of a pie chart. In **(B)**, the expression of different markers (X-axis) for the different clusters (Y-axis) are depicted in a heatmap ranging from blue (low) to red (high). The differential abundance for comparing MMF-IS to EVR-IS at M12 is depicted in **(C)**, with the P-value depicted as -log10 (false discovery rate, FDR) value on the Y-axis and log fold change on the X-axis. The green colored clusters are significantly different between the 2 treatment arms with the cluster number depicted. In the table given in **(D)**, the expression of different markers for the significantly different clusters are depicted using + (high/present) and – (low/absent).

FlowSOM analysis resulted in a minimal spanning tree consisting of 24 different cell clusters (**Figure 2A**). Translating the expression of different markers to a heatmap, revealed 10 of them containing CD4+ T cells, 9 containing CD8+ T cells, 1 CD4+CD8+ T cells and 4 CD4-CD8- T cells (**Figure 2B**). Three clusters were significantly different comparing MMF-IS to EVR-IS at M12, i.e. cluster 11, 6 and 2 (volcano plot in **Figure 2C**). As the FlowSOM algorithm groups cells by assessing their entire range of marker expression, its resulting clusters do not correspond exactly to populations identified through traditional gating. FlowSOM captures continuous marker profiles, while traditional gating relies on a simple binary (positive/negative) classification of specific markers. Furthermore, effectively evaluating all the markers within this panel using traditional gating would require generating multiple two-dimensional plots and complex Boolean (and/or/not) logic. Nevertheless, the increase in Tregs for EVR-IS at M12, based on the traditional gating was reflected in cluster 11 as it corresponded to Tregs (2-fold lower in MMF-IS, P<0.01) based on expression profiles of FOXP3, CD25 and CD127 (**Figure 2D**). Cluster 6 consisted of CD4+ T cells with characteristics of less differentiated (CD27dimCD28+) Tem co-expressing the proliferation marker Ki-67 (1.9-fold lower in MMF-IS, P = 0.04; **Figure 2D**) and cluster 2 contained CD8+ T cells with a terminal differentiated Tem profile co-expressing the transcription factor Tbet (2.4-fold higher in MMF-IS, P = 0.04; **Figure 2D**).

### 3.4 Decline of polyfunctional donor antigen-specific alloreactive CD4+ T cells but stable number of virus-reactive CD4+ T cells

**Figure 3:**
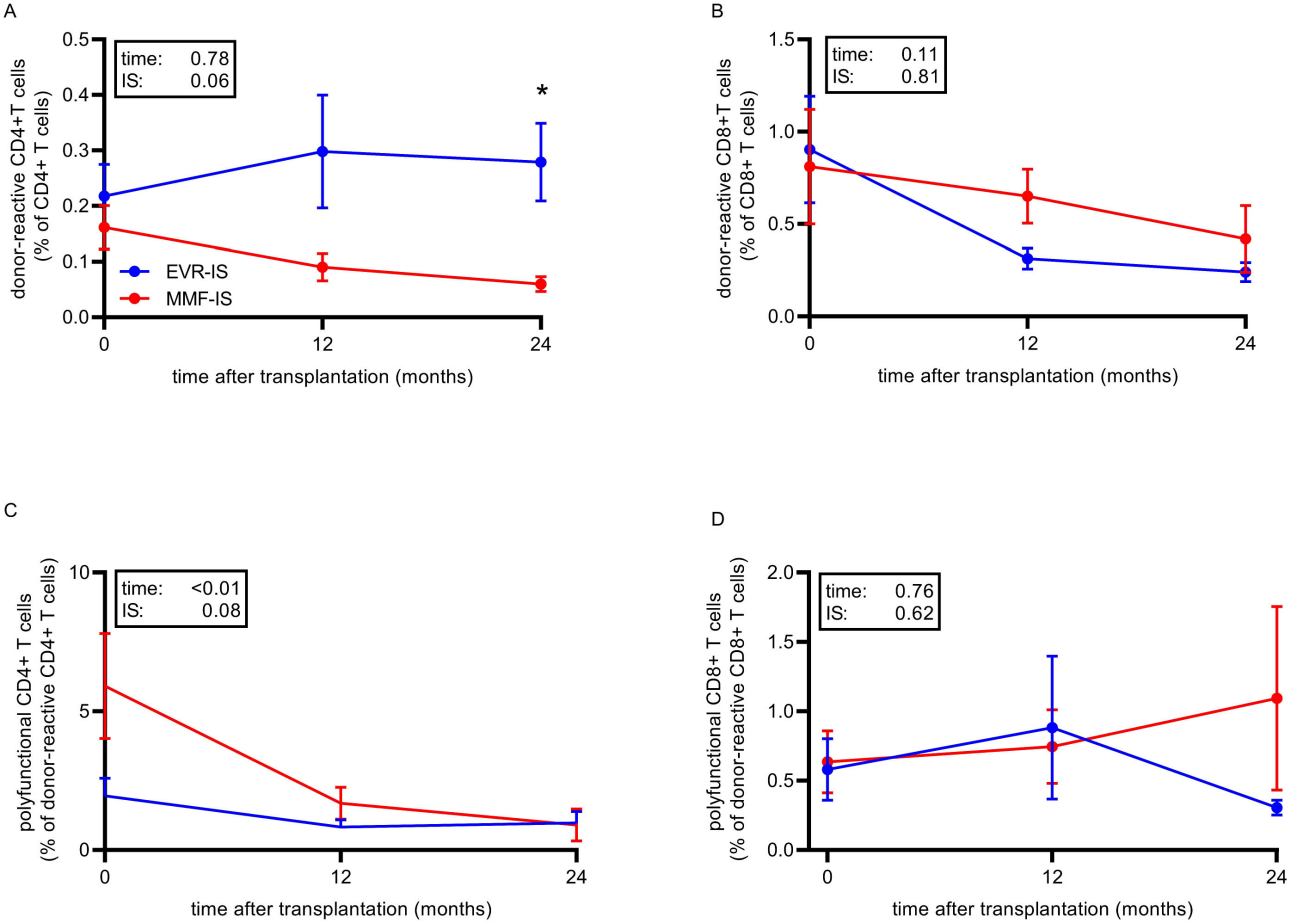
Kinetics of alloreactive CD4+ and CD8+ T cells. Alloreactive T cells were characterized in detail for phenotypic as well as functional characteristics in 18 kidney transplant recipients (N = 8 MMF-IS and N = 10 EVR-IS) prior to and at M12 (N = 8 MMF-IS and N = 9 EVR-IS) at M24 (N = 4 MMF-IS and M = 5 EVR-IS) after transplantation. Proportions of alloreactive CD4+ **(A)** and CD8+ **(B)** T cells are depicted upon short-term stimulation with CD3-depleted donor-cells. Subsequently, proportions of polyfunctional, i.e. those producing 2 or more pro-inflammatory cytokines, alloreactive CD4+ **(C)** and CD8+ **(D)** T cells are given for 17 kidney transplant recipients (N = 7 MMF-IS and N = 10 EVR-IS) prior to and at M12 (N = 9 MMF-IS and N = 10 EVR-IS) and at M24 (N = 4 MMF-IS and 7 EVR-IS) after transplantation. Mean ± SEM are depicted and blue lines represent the kinetics for kidney transplant recipients receiving EVR-based IS whereas those receiving MMF-based IS are depicted in red. Mixed effect analysis with multiple comparison test was used to determine effect of time and treatment arm (IS) on the kinetics of T cell subsets (P-values listed in square) and to compare the difference between both treatment arms for each time-point. * represents a P-value< 0.05.

Comparing proportions of donor antigen-specific alloreactive CD4+ T cells after transplantation tended to reveal an effect of treatment arm (P = 0.06). Proportions were higher for EVR-IS when compared to MMF-IS at M12 (P = 0.06) and M24 (P = 0.03) (**Figure 3A**). Average proportions at M12 and M24 amounted to 0.30 ± 0.10% and 0.28 ± 0.07% versus 0.09 ± 0.03% and 0.06 ± 0.01% for EVR-IS and MMF-IS, respectively. The proportion of polyfunctional alloreactive CD4+ T cells declined significantly over time for both treatment arms (P<0.01). The average decline amounted to 57% and 71% for EVR-IS (P = 0.08) and MMF-IS (P = 0.03), respectively (**Figure 3C**) whereas the proportion of polyfunctional virus-reactive CD4+ T cells remained similar (**Supplementary Figure 6A**). Average proportions of polyfunctional CD4+ T cells declined within the donor antigen-specific alloreactive T cell fraction from 2.0 ± 0.6% and 5.9 ± 1.9% prior to transplantation to 0.8 ± 0.3% and 1.7 ± 0.6% 12 months after transplantation for EVR-IS and MMF-IS, respectively.

Interestingly, proportions of alloreactive CD8+ Teff decreased within the first 12 months after transplantation for EVR-IS (P = 0.04) whereas this decline was delayed for MMF-IS (**Figure 3B**). Average percentages declined in the EVR-IS group by 60% (from 0.9 ± 0.3% to 0.3 ± 0.1%) versus 24% (from 0.8 ± 0.3% to 0.7 ± 0.1%) in the MMF-IS group. No specific differences in patterns of cytokine secreting donor antigen-specific alloreactive CD8+ T cells between the EVR-IS and MMF-IS group were noted in the course after transplantation (**Figure 3D**). Like observed for CD4+ T cells, the percentage of polyfunctional virus-reactive CD8+ T cells remained stable over time after transplantation (**Supplementary Figure 6B**).

### 3.5 Everolimus is associated with an increase of alloreactive Tregs

**Figure 4:**
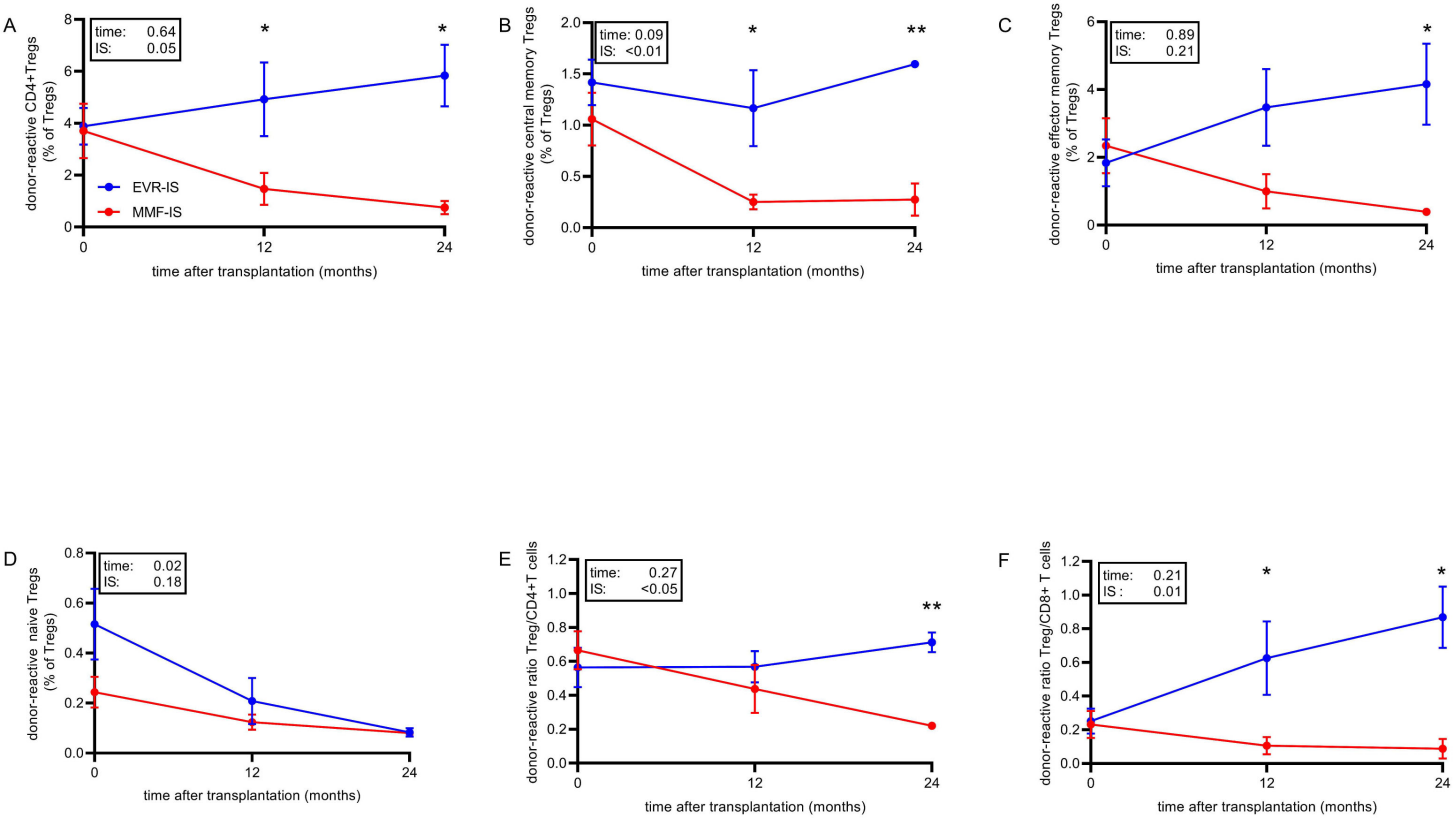
Kinetics of alloreactive CD4+ regulatory T cells. Proportions of donor antigen-specific alloreactive CD4+ regulatory T cells **(A)** are depicted upon short-term stimulation with CD3-depleted donor-cells. Donor antigen-specific alloreactive Tregs were subsequently dissected into central memory **(B)**, effector memory **(C)** and naïve **(D)** Tregs using expression of CD45RA and CCR7. On the X-axis, the different time-points are depicted whereas on the Y-axis, the fraction of a specific subset is depicted as a percentage of total Tregs. Next, the ratio of donor antigen-specific alloreactive regulatory T cells over CD4+ T cells is given in **(E)** whereas that over CD8+ T cells is given in **(F)** Mean ± SEM are depicted and blue lines represent the kinetics for kidney transplant recipients receiving EVR-based IS (N = 10 prior to and N = 9 and N = 5 at M12 and M24 after transplantation, respectively) whereas those receiving MMF-based IS (N = 8 prior to and N = 8 and N = 4 after transplantation, respectively) are depicted in red. Mixed effect analysis with multiple comparison test was used to determine effect of time and treatment arm (IS) on the kinetics of T cell subsets (P-values listed in square) and to compare the difference between both treatment arms for each time-point. * represents a P-value < 0.05 and ** a P-value <0.01.

Alloreactive Tregs increased only for the EVR-IS group after transplantation (**Figure 4A**). Average percentages of alloreactive Tregs within the total Treg population increased for EVR-IS, i.e. 4.92 ± 1.42% and 5.84 ± 1.19% whereas the reversed was observed for the MMF-IS group (1.48 ± 0.61% and 0.75 ± 0.26%) at M12 and M24, respectively (**Figure 4A**). A detailed phenotypic characterization of these Tregs, revealed an increase in central memory (**Figure 4B**) and effector memory (**Figure 4C**) donor antigen-specific alloreactive Tregs for EVR-IS compared to MMF-IS. Proportions of naïve donor antigen-specific alloreactive Tregs declined for both treatment regimens (**Figure 4D**).

A significant higher ratio of donor antigen-specific alloreactive Tregs over CD4+ T cells was observed for EVR-IS when compared to MMF-IS (0.71 ± 0.06 versus 0.22 ± 0.02, p<0.01) at M24 after transplantation (**Figure 4E**). An even more striking effect was observed between the two treatment arms when the ratio of donor antigen-specific alloreactive Tregs over CD8+ T cells was compared (**Figure 4F**). At M12 and M24 the average ratios amounted to 0.63 ± 0.21 and 0.87 ± 0.18 versus 0.11 ± 0.05 and 0.09 ± 0.06 for EVR-IS and MMF-IS, respectively (P-value for effect of treatment-arm = 0.01).

### 3.6 Suppressive capacity of Tregs

**Figure 5:**
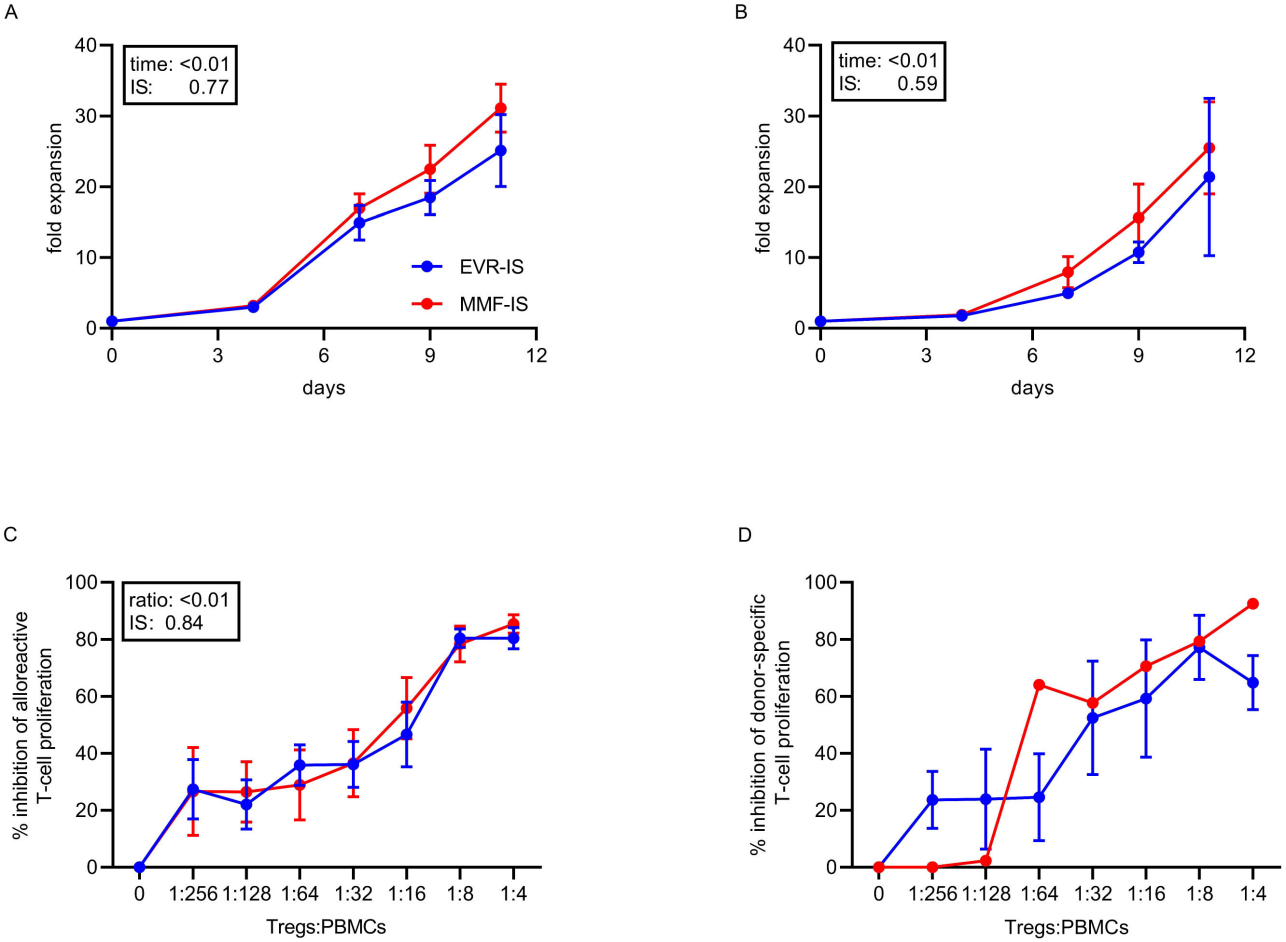
Suppressive potential of Tregs. Fold expansion for αCD3/αCD28-induced (polyclonal) [**(A)**, N = 10, 5 EVR-IS and 5 MMF-IS] and donor antigen-induced [**(B)**, N = 6, N = 4 EVR-IS and N = 2 MMF-IS] expanded FACS-sorted Tregs is depicted, by dividing the number of Tregs at a certain time over that at day 0. In **(C)**, inhibition of alloantigen-induced proliferation in presence of polyclonal-expanded Tregs, normalized to proliferation in absence of Tregs is depicted (N = 13, N = 8 EVR-IS and N = 5 MMF-IS). Blue lines represent the dose-response curve for kidney transplant recipients receiving EVR-based IS whereas those receiving MMF-based IS are depicted in red. In **(D)**, inhibition of donor antigen-induced proliferation in presence of donor antigen-expanded Tregs was depicted for EVR-IS (blue line, N = 4) and MMF-IS (red line, N = 2, due to the low yield of sorted Tregs obtained for expansion) **(D)**. Mean ± SEM are depicted. Mixed effect analysis with multiple comparison test was used to determine effect of time **(A, B)** or ratio of Tregs: PBMCs **(C)** and treatment arm (IS) on expansion **(A, B)** or inhibition of proliferation **(C)**. P-values listed in square.

Highly purified Tregs were polyclonal (**Figure 5A**) or donor antigen-specific expanded (**Figure 5B**) with similar efficacy between both treatment arms. Average ± SEM fold expansion amounted to 28.1 ± 3.1 and 23.4 ± 6 for polyclonal and donor antigen-specific expansion, respectively. Donor antigen-induced proliferation was equally well inhibited in a dose-response manner (P<0.01) by polyclonal Tregs from both EVR-IS and MMF-IS treated recipients (**Figure 5C**; P = 0.84 for IS). Donor antigen-specific expanded Tregs of kidney transplant recipients on EVR-IS were also able to inhibit donor antigen-induced proliferation with no difference when compared to donor antigen-specific expanded Tregs of kidney transplant recipients on MMF-IS (**Figure 5D**).

## 4 Discussion

In this study the differential impact of everolimus, combined with low dose tacrolimus and prednisolone compared to MMF combined with standard dose tacrolimus and prednisolone on circulating T cell subsets after kidney transplantation was investigated. The results show that the EVR-IS group had relative stable proportions of effector memory T cells but a significant increase of Tregs over time. Instead, an MMF-IS regimen leads to a decrease in effector memory T cells, particularly among the CD4+ T cells accompanied by a decrease in natural Tregs. Regarding the donor antigen-specific alloreactivity, a decrease in polyfunctional CD4+ effector T cells was observed over time after transplantation. Although based on a sub study, consisting of a smaller cohort of kidney transplant recipients, the most striking differences between both regimens were a significant increase in donor antigen-specific alloreactive Tregs, a more or less stable donor antigen-specific alloreactive CD4+ effector T cell compartment and a more rapid decline in donor antigen-specific alloreactive CD8+ effector T cells in the EVR-IS treated recipients compared to those on MMF-IS. This resulted in significant higher ratios of donor antigen-specific alloreactive Treg/Teff for EVR-IS treated patients.

In line with the current results, a previous large study on the impact of MMF-IS on T cells showed that the effector CD4+ T cells decreased within one year after transplantation while no impact was observed on total number of CD4+ and CD8+ T cells and thymic output of naïve T cells (19). Overall, kidney transplantation does not change the features of premature T cell aging of patients with end-stage chronic kidney disease (e.g. decreased thymic output and increased T cell differentiation) (20). A similar conclusion can be drawn for the EVR-IS treated recipients over a period of 24 months after kidney transplantation.

A major difference between the EVR-IS and MMF-IS groups was the significant expansion of natural Tregs in the former group. The first identified mTORi rapamycin had the capacity to induce Treg expansion *in vitro (*8, 21, 22) but the data on Tregs in humans treated with rapamycin were equivocal (21, 23). While rapamycin-treated compared to CNI-treated patients showed significant increased numbers of circulating natural Tregs, there was no difference between rapamycin or MMF-treated patients indicating that CNI use can lead to a decrease in Tregs (24). Furthermore, T cell depleting induction therapy and different criteria for identifying circulating Tregs have been used in studies evaluating mTORi and Tregs after transplantation (21, 24). Of note, in this study everolimus was combined with low-dose tacrolimus and shows that low dose tacrolimus in combination with everolimus still allowed for Treg expansion. CNI inhibitors like tacrolimus, are known for interfering in calcineurin-dependent IL-2 production, an important factor for Treg expansion and function and therefore impair Treg expansion (25). Surprisingly, tacrolimus trough levels did not correlate with Treg proportions at M12 in the MMF-IS group. Moreover, Treg expansion occurred exclusively in the EVR-IS group, which aligns with numerous *in vitro* and *in vivo* studies showing mTORi to facilitate expansion of Tregs (22, 26, 27). Because high tacrolimus trough levels were absent in the EVR-IS group, it remains unclear whether a low dose of tacrolimus is necessary to permit expansion of Tregs. Natural Tregs were identified by high expression of CD25 in combination with low CD127 expression. Previously, we have shown that cells within this population of CD4+ T cells all express a demethylated TSDR which is associated with constitutive expression of transcription factor FOXP3, the hallmark of natural Tregs (12, 28). In addition, this population of cells contained alloreactive Tregs and had a very high potential to inhibit proliferation of effector T cells. Markers of Tregs like CD25 and FOXP3 may be upregulated and CD127 downregulated shortly after activation of effector T cells which obscures the differentiation with natural Tregs. For this reason, we successfully developed a protocol to stain the cell surface for CD25 and CD127 prior to antigen stimulation which allowed for reliable identification of donor antigen-specific alloreactive Tregs by CD137. This clear discrimination of Tregs from effector cells following a brief stimulation greatly facilitates the evaluation of different antigen-reactive T cell fractions using activation-induced markers (AIMs) such as CD137.

After transplantation the proportion of both donor antigen-specific alloreactive CD4+ and CD8+ T cells declined for MMF-IS treated older kidney transplant recipients which is in line with the results of a previous study in both young and older kidney transplant recipients treated with maintenance IS consisting of MMF and tacrolimus (14, 29). For EVR-IS, donor antigen-specific alloreactive CD4+ T cells remained more or less stable whereas donor antigen-specific alloreactive CD8+ T cells declined more rapidly compared to MMF-IS. Polyfunctional donor antigen-specific alloreactive CD4+ T cells declined for both treatment regimens. This cell population is associated with the risk for acute rejection and the loss of these cells with donor-specific hypo-responsiveness (13, 14, 30). An interesting observation was that the frequency of virus-reactive polyfunctional T cells remained unchanged after transplantation in both treatment arms corresponding to a similar incidence of CMV-infections. In line with this finding, a previous study showed that kidney transplant recipients treated with rapamycin had a similar frequency of CMV-specific CD4+ T cells as the MMF-treated recipients (31). Also, in the recently published OPTIMIZE trial no difference in CMV- infections between treatment arms was observed (6). We did not analyze proportions of virus-reactive Tregs and calculate virus-reactive effector to Treg ratios which might have provided more mechanistic insight. However, our data indicate that both regimens allow for effective control of virus-infections by virus-reactive effector T cells. Not only the total proportion of Tregs but also the fraction of donor antigen-specific alloreactive CD137+ Tregs increased during treatment with EVR-IS while these cells decreased in the MMF-IS group, leading to a major increase in donor antigen-specific alloreactive Treg: Teff ratio for EVR-IS compared to MMF-IS for both effector CD4+ but especially for CD8+ T cells. Analysis of isolated Tregs showed excellent suppressive function for alloreactive T cells, irrespective of the IS regimen.

These preliminary findings indicate that functional donor antigen-specific alloreactive Tregs significantly expand in time after transplantation using EVR-based IS and that these effectively control Teff. This may be the mechanistic explanation for the clinical observation that low maintenance trough levels of tacrolimus can be used safely in combination with everolimus (3). Under MMF-IS, donor antigen-specific alloreactive Teff are deleted via activation-induced cell death (14, 30), controlling the alloimmune response in a different manner. The incidence of acute rejection is similar low in this cohort of older kidney transplant recipients, indicating that both treatment regimens may successfully promote allograft survival. Also, in the completed OPTIMIZE trial no difference was found in rejection episodes between the two regimens (6). The initial observations from the small scale immunological sub studies performed here, require validation in a larger cohort of elderly kidney transplant recipients on EVR-IS and MMF-IS. Moreover, a longer follow-up is warranted to evaluate whether these two regimens remain similar with respect to long-term results.

## 5 Conclusion

In conclusion, this study demonstrated that everolimus-based low-dose tacrolimus and MMF-based standard-dose tacrolimus regimens exploit different mechanisms to control the alloimmune response. This distinct modulation, requiring validation in a larger cohort, may offer a powerful framework for tailoring risk-stratified immunosuppression in older kidney transplant recipients optimizing kidney allograft survival.

## Data Availability

The original contributions presented in the study are included in the article/**Supplementary Material**. Further inquiries can be directed to the corresponding author.

## References

[1] Hariharan S Israni AK Danovitch G . Long-term survival after kidney transplantation. N Engl J Med. (2021) 385:729–43. doi: 10.1093/med/9780197697320.003.0001 34407344

[2] Naesens M Kuypers DR Sarwal M . Calcineurin inhibitor nephrotoxicity. Clin J Am Soc Nephrol. (2009) 4:481–508. doi: 10.2215/cjn.04800908 19218475

[3] Berger SP Sommerer C Witzke O Tedesco H Chadban S Mulgaonkar S . Two-year outcomes in de novo renal transplant recipients receiving everolimus-facilitated calcineurin inhibitor reduction regimen from the TRANSFORM study. Am J Transplant. (2019) 19:3018–34. doi: 10.1111/ajt.15480 31152476

[4] Takada Y Tanabe T Sasaki H Tsujimoto T Hotta K Okada K . Kidney donor age of 50 years or above is a risk factor for calcineurin inhibitor-induced nephrotoxicity. Clin Transplant. (2024) 38:e15196. doi: 10.1111/ctr.15196 37975424

[5] de Boer SE Sanders JSF Bemelman FJ Betjes MGH Burgerhof JGM Hilbrands L . Rationale and design of the OPTIMIZE trial: OPen label multicenter randomized trial comparing standard IMmunosuppression with tacrolimus and mycophenolate mofetil with a low exposure tacrolimus regimen in combination with everolimus in de novo renal transplantation in elderly patients. BMC Nephrol. (2021) 22:208. doi: 10.1186/s12882-021-02409-8 34078323 PMC8172178

[6] Sanders JF de Boer SE Jonker J Bemelman FJ Betjes MGH de Vries APJ . Immunosuppression in older kidney transplant recipients: A randomized controlled trial. J Am Soc Nephrol. (2025) 37(4):814–24. doi: 10.1681/ASN.0000000924 PMC13065175 41201871

[7] Wullschleger S Loewith R Hall MN . TOR signaling in growth and metabolism. Cell. (2006) 124:471–84. doi: 10.1016/j.cell.2006.01.016 16469695

[8] Coenen JJ Koenen HJ van Rijssen E Hilbrands LB Joosten I . Rapamycin, and not cyclosporin A, preserves the highly suppressive CD27+ subset of human CD4+CD25+ regulatory T cells. Blood. (2006) 107:1018–23. doi: 10.1182/blood-2005-07-3032 16210336

[9] Litjens NH van de Wetering J van Besouw NM Betjes MG . The human alloreactive CD4+ T-cell repertoire is biased to a Th17 response and the frequency is inversely related to the number of HLA class II mismatches. Blood. (2009) 114:3947–55. doi: 10.1182/blood-2009-03-211896 19713464

[10] Litjens NH de Wit EA Baan CC Betjes MG . Activation-induced CD137 is a fast assay for identification and multi-parameter flow cytometric analysis of alloreactive T cells. Clin Exp Immunol. (2013) 174:179–91. doi: 10.1111/cei.12152 23750604 PMC3784225

[11] Reiss S Baxter AE Cirelli KM Dan JM Morou A Daigneault A . Comparative analysis of activation induced marker (AIM) assays for sensitive identification of antigen-specific CD4 T cells. PLoS One. (2017) 12:e0186998. doi: 10.1371/journal.pone.0186998 29065175 PMC5655442

[12] Litjens NH Boer K Betjes MG . Identification of circulating human antigen-reactive CD4+ FOXP3+ natural regulatory T cells. J Immunol. (2012) 188:1083–90. doi: 10.4049/jimmunol.1101974 22190183

[13] Litjens NHR van der List ACJ Klepper M Prevoo F Boer K Hesselink DA . Polyfunctional donor-reactive T cells are associated with acute T-cell-mediated rejection of the kidney transplant. Clin Exp Immunol. (2023) 213:371–83. doi: 10.1093/cei/uxad041 37070703 PMC10571010

[14] van der List ACJ Litjens NHR Klepper M Prevoo F Betjes MGH . Progressive loss of donor-reactive CD4(+) effector memory T cells due to apoptosis underlies donor-specific hyporesponsiveness in stable renal transplant recipients. J Immunol. (2022) 209:1389–400. doi: 10.4049/jimmunol.2200352 36165198

[15] Betjes MGH Klepper M Smits G van der Valk E van der List ACJ Litjens NHR . Recognition of different subsets of alloreactive T cells by activation-induced markers. Transpl Immunol. (2025) 90:102227. doi: 10.1016/j.trim.2025.102227 40204006

[16] Wijngaarden LH Taselaar AE Nuijten F van der Harst E Klaassen RA Kuijper TM . T and B cell composition and cytokine producing capacity before and after bariatric surgery. Front Immunol. (2022) 13:888278. doi: 10.3389/fimmu.2022.888278 35860273 PMC9289114

[17] Van Gassen S Callebaut B Van Helden MJ Lambrecht BN Demeester P Dhaene T . FlowSOM: Using self-organizing maps for visualization and interpretation of cytometry data. Cytometry A. (2015) 87:636–47. doi: 10.1007/978-3-540-92910-9_19 25573116

[18] Litjens NH Boer K Zuijderwijk JM Klepper M Peeters AM Prens EP . Allogeneic mature human dendritic cells generate superior alloreactive regulatory T cells in the presence of IL-15. J Immunol. (2015) 194:5282–93. doi: 10.4049/jimmunol.1402827 25917092

[19] Meijers RW Litjens NH de Wit EA Langerak AW Baan CC Betjes MG . Uremia-associated immunological aging is stably imprinted in the T-cell system and not reversed by kidney transplantation. Transpl Int. (2014) 27:1272–84. doi: 10.1111/tri.12416 25082296

[20] Betjes MG Langerak AW van der Spek A de Wit EA Litjens NH . Premature aging of circulating T cells in patients with end-stage renal disease. Kidney Int. (2011) 80:208–17. doi: 10.1038/ki.2011.110 21525849

[21] Shan J Feng L Li Y Sun G Chen X Chen P . The effects of rapamycin on regulatory T cells: Its potential time-dependent role in inducing transplant tolerance. Immunol Lett. (2014) 162:74–86. doi: 10.1016/j.imlet.2014.07.006 25149861

[22] Battaglia M Stabilini A Roncarolo M-G . Rapamycin selectively expands CD4+CD25+FoxP3+ regulatory T cells. Blood. (2005) 105:4743–8. doi: 10.1182/blood-2004-10-3932 15746082

[23] Libetta C Esposito P Gregorini M Margiotta E Martinelli C Borettaz I . Sirolimus vs cyclosporine after induction with basiliximab does not promote regulatory T cell expansion in de novo kidney transplantation: Results from a single-center randomized trial. Transpl Immunol. (2015) 33:117–24. doi: 10.1016/j.trim.2015.07.005 26220254

[24] Miroux C Morales O Ghazal K Othman SB de Launoit Y PancrÃ© V . *In vitro* effects of cyclosporine A and tacrolimus on regulatory T-cell proliferation and function. Transplantation. (2012) 94:123–31. doi: 10.1097/tp.0b013e3182590d8f 22743548

[25] Zeiser R Nguyen VH Beilhack A Buess M Schulz S Baker J . Inhibition of CD4+CD25+ regulatory T-cell function by calcineurin-dependent interleukin-2 production. Blood. (2006) 108:390–9. doi: 10.1182/blood-2006-01-0329 16522809 PMC1895845

[26] Levitsky J Miller J Huang X Gallon L Leventhal JR Mathew JM . Immunoregulatory effects of everolimus on *in vitro* alloimmune responses. PLoS One. (2016) 11:e0156535. doi: 10.1371/journal.pone.0156535 27275747 PMC4898829

[27] Zeiser R Leveson-Gower DB Zambricki EA Kambham N Beilhack A Loh J . Differential impact of mammalian target of rapamycin inhibition on CD4+CD25+Foxp3+ regulatory T cells compared with conventional CD4+ T cells. Blood. (2008) 111:453–62. doi: 10.1182/blood-2007-06-094482 17967941 PMC2200823

[28] Litjens NH Boer K Zuijderwijk JM Klepper M Peeters AM Verschoor W . Natural regulatory T cells from patients with end-stage renal disease can be used for large-scale generation of highly suppressive alloantigen-specific Tregs. Kidney Int. (2017) 91:1203–13. doi: 10.1016/j.kint.2016.09.043 27988212

[29] Litjens NHR van der List ACJ Klepper M Reijerkerk D Prevoo F Betjes MGH . Older age is associated with a distinct and marked reduction of functionality of both alloreactive CD4+ and CD8+ T cells. Front Immunol. (2024) 15:1406716. doi: 10.3389/fimmu.2024.1406716 39044836 PMC11263037

[30] van der List ACJ Litjens NHR Brouwer RWW Klepper M den Dekker AT van Ijcken WFJ . Single-cell RNA sequencing of donor-reactive T cells reveals role of apoptosis in donor-specific hyporesponsiveness of kidney transplant recipients. Int J Mol Sci. (2023) 24:1–17. doi: 10.3390/ijms241914463 37833911 PMC10572284

[31] Hauser IA Marx S Sommerer C Suwelack B Dragun D Witzke O . Effect of everolimus-based drug regimens on CMV-specific T-cell functionality after renal transplantation: 12-month ATHENA subcohort-study results. Eur J Immunol. (2021) 51:943–55. doi: 10.1002/eji.202048855 33306229

