# Supplementary material for: Everolimus-based immunosuppression induces alloreactive regulatory T cell expansion combined with loss of alloreactive effector T cells
Source: Front Immunol. 2026 Aug 3;17:1904559. doi: 10.3389/fimmu.2026.1904559 (PMC13478954; doi:10.3389/fimmu.2026.1904559)
Supplement: Supplementary file 1 [file DataSheet1.pdf]

Supplementary Table 1 Analysis of T cell phenotype

Marker	Fluorochrome	Titer (μL) *	Company	Clone
Cell surface staining				
Brilliant stain plus buffer		10	BD**	none
CD3	AF700	0.25	Biolegend***	HIT3a
CD4	PerCPCy5.5	1	BD	RPA-T4
CD8	BV510	1	BioLegend	RPA-T8
CD25	APC	0.25	BioLegend	BC96
CD127	PE-Dazzle594	0.5	BioLegend	A019D5
CD45RA	BV650	0.5	BioLegend	HI100
CCR7	BV421	0.5	Biolegend	G043H7
CD28	BV785	1	Biolegend	CD28.2
CD27	BV605	2.5	BD	L128
CD31	PE	0.25	Biolegend	WM59
CD14	APC-H7	0.125	BD	MφP9
CD19	APC-H7	0.5	BD	SJ25C1
CD56	APC-Cy7	0.5	BioLegend	HCD56
FVS780		0.25**	BD	none
Intracellular staining				
FOXP3	AF488	5	Biolegend	259D
Ki-67	BV711	1	Biolegend	4B10
Tbet	PE-Cy7	0.125	Biolegend	Ki-67

* Staining performed in total volume of 50 μL

** staining performed before rest of surface staining 0.25 μL/1 mL

Supplementary Table 2A Baseline study population characteristics functional analysis donor-antigen-specific alloreactive T cells

	EVR-IS	MMF-IS	P-value
Number of individuals	11	9	
Age acceptor in years, median [IQR]*	71 (67-74)	68 (66-75)	0.33
Male, n(%)	9 (82)	4 (44)	0.16
Donor age in years, median [IQR] *	67 (59-71)	68 (56-70)	0.8
Donor Male, n(%)	4 (36)	6 (67)	0.37
First kidney transplant, n(%)			>0.99
Type of transplantation	4 (36)	3 (33)	>0.99
Living donor, n(%)	7 (64)	6 (67)	
Deceased donor, n(%)			
Number of mismatches HLA, median [IQR]	2 (1-3)	3 (2-3)	
HLA class I	2 (1-2)	1 (1-2)	0.62
HLA class II			0.97
Renal replacement therapy, n(%)	2 (18)	2 (22)	>0.99
No (pre-emptive)	9 (82)	7 (78)	
Dialysis			
CMV serostatus acceptor*<br>*Seropositive, n(%)*<br>*Seronegative, n(%)*	 6 (55)<br>5 (45)	 5 (56)<br>4 (44)	>0.99

* *at time of transplantation*

Supplementary Table 2B Baseline study population characteristics functional analysis virus-specific alloreactive T cells

	EVR-IS	MMF-IS	P-value
Number of individuals	12	13	
Age acceptor in years, median [IQR]*	71 (65-76)	71 (69-72)	0.75
Male, n(%)	7 (58)	9 (69)	0.69
Donor age in years, median [IQR] *	69 (55-74)	68 (59-73)	0.75
Donor Male, n(%)	6 (50)	7 (54)	>0.99
First kidney transplant, n(%)	12 (100)	13 (100)	>0.99
Type of transplantation			>0.99
Living donor, n(%)	8 (67)	8 (62)	
Deceased donor, n(%)	4 (33)	5 (38)	
Number of mismatches HLA, median [IQR]			
HLA class I	3 (1-3)	2 (2-4)	0.36
HLA class II	2 (1-2)	1 (1-2)	0.87
Renal replacement therapy, n(%)			>0.99
No (pre-emptive)	7 (58)	7 (54)	
Dialysis	5 (42)	6 (46)	
CMV serostatus acceptor*			>0.99
Seropositive, n(%)	9 (75)	10 (77)	
Seronegative, n(%)	3 (25)	3 (23)	

* *at time of transplantation*

Supplementary Table 2C Baseline study population characteristics phenotypic analysis donor antigen-specific alloreactive effector and regulatory T cells

	EVR-IS	MMF-IS	P-value
Number of individuals	10	10	
Age acceptor in years, median [IQR]*	71 (67-77)	69 (66-72)	0.12
Male, n(%)	6 (60)	5 (50)	>0.99
Donor age in years, median [IQR] *	64 (44-73)	67 (59-69)	0.37
Donor Male, n(%)	7 (70)	4 (40)	0.37
First kidney transplant, n(%)	10 (100)	10 (100)	>0.99
Type of transplantation			>0.99
Living donor, n(%)	4 (40)	4 (40)	
Deceased donor, n(%)	6 (60)	6 (60)	
Number of mismatches HLA, median [IQR]			
HLA class I	3 (2-3)	3 (2-3)	0.84
HLA class II	1 (1-1)	1 (1-2)	0.15
Renal replacement therapy, n(%)			>0.99
No (pre-emptive)	4 (40)	4 (40)	
Dialysis	6 (60)	6 (60)	
CMV serostatus acceptor*			>0.99
Seropositive, n(%)	8 (80)	7 (70)	
Seronegative, n(%)	2 (20)	3 (30)	

* *at time of transplantation*

Supplementary Table 2D Baseline study population characteristics for analysis of Treg function

	EVR-IS	MMF-IS	P-value
Number of individuals	5	5	
Age acceptor in years, median [IQR]*	69 (67-72)	70 (68-71)	>0.99
Male, n(%)	4 (80)	3 (60)	>0.99
Donor age in years, median [IQR] *	66 (58-68)	68 (61-69)	0.6
Donor Male, n(%)	2 (40)	3 (60)	>0.99
First kidney transplant, n(%)	4 (80)	5 (100)	>0.99
Type of transplantation			>0.99
Living donor, n(%)	3 (60)	3 (60)	
Deceased donor, n(%)	2 (40)	2 (40)	
Number of mismatches HLA, median [IQR]			
HLA class I	2 (2-4)	3 (2-4)	0.54
HLA class II	2 (1-2)	1 (1-2)	0.58
Renal replacement therapy, n(%)			>0.99
No (pre-emptive)	2 (40)	2 (40)	
Dialysis	3 (60)	3 (60)	
CMV serostatus acceptor*			0.21
Seropositive, n(%)	4 (80)	1 (20)	
Seronegative, n(%)	1 (20)	4 (80)	

* *at time of transplantation*

Supplementary Table 3 Analysis of polyfunctional antigen-reactive T cells

Marker	Fluorochrome	Titer (μL) *	Company	Clone
Present during stimulation				
CD107a	FITC	5	Biolegend	H4A3
Cell surface staining				
Brilliant stain plus buffer		10	BD	none
CD3	AlexaFluor 700	0.25	Biolegend	HIT3a
CD4	PerCPCy5.5	1	BD	RPA-T4
CD8	BV510	1	BioLegend	RPA-T8
CD45RA	BV650	0.5	BioLegend	HI100
CCR7	BV421	0.5	Biolegend	G043H7
CD14	APC-H7	0.125	BD	MφP9
CD19	APC-H7	0.5	BD	SJ25C1
CD56	APC-Cy7	0.5	BioLegend	HCD56
FVS780		0.25**	BD	none
Intracellular staining				
Brilliant stain plus buffer		10	BD	none
CD137	APC	2	BD	4B4-1
IL-2	BV785	0.25	Biolegend	MQ1-17H12
IFN-γ	BV711	0.5	Biolegend	4S.B3
TNF-α	BV605	1	Biolegend	Mab11

* Staining performed in total volume of 50 μL

** staining performed before rest of surface staining 0.25 μL/1 mL

Supplementary Table 4 Analysis of phenotype of alloreactive T cells

Marker	Fluorochrome	Titer (μL) *	Company	Clone
Cell surface staining				
Brilliant stain plus buffer		10	BD**	none
CD3	AF700	0.25	Biolegend***	HIT3a
CD4	PerCPCy5.5	1	BD	RPA-T4
CD8	BV510	1	BioLegend	RPA-T8
CD14	APC-H7	0.125	BD	MφP9
CD19	APC-H7	0.5	BD	SJ25C1
CD56	APC-Cy7	0.5	BioLegend	HCD56
FVS780		0.25***	BD	none
CD45RA	BV650	0.5	BioLegend	HI100
CCR7	BV421	0.5	Biolegend	G043H7
CD28	BV785	1	Biolegend	CD28.2
CD27	BV605	2.5	BD	L128
CD137	APC	2	BD	4B4-1

* Staining performed in total volume of 50 μL

** staining performed before rest of surface staining 0.25 μL/1 mL

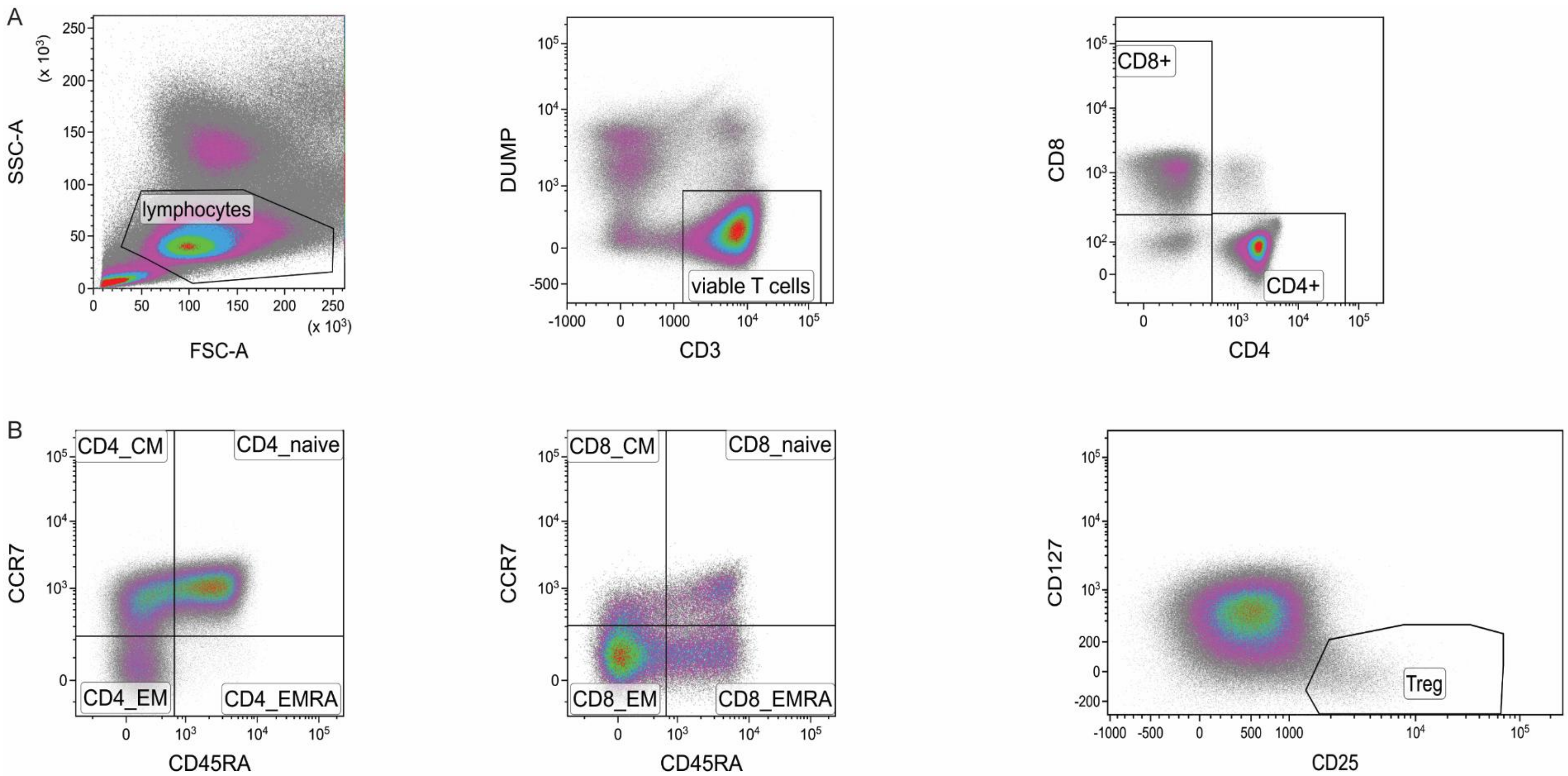

Supplementary Figure 1 Analysis of circulating T cell subsets

Representative example of the gating strategy applied for analysis of circulating T cell subsets is depicted.
In the top panel (**A**), lymphocytes are selected (first plot) based on forward-sideward characteristics. These lymphocytes are than plotted for CD3 (X-axis) and using a dumpchannel, consisting of antibodies directed against, CD14, CD19, CD56 and the live-dead stain FVS780, viable T cells are selected (second plot). In the third plot, CD4 and CD8+ T cells are identified. In the lower panel (**B**), the different CD4+ (first plot) and CD8+ (second plot) T cell subsets are identified using CD45RA on X-axis and CCR7 on Y-axis. Naive T cells are CD45RA+ and CCR7+, central memory T cells (CM) are CD45RA-CCR7+, effector memory T cells (EM) are CD45RA-CCR7- and effector memory re-expressing CD45RA (EMRA) are CD45RA+ aand CCR7-.
In the third plot, regulatory CD4+ T cells are identified using CD25 (X-axis) and CD127 (Y-axis).

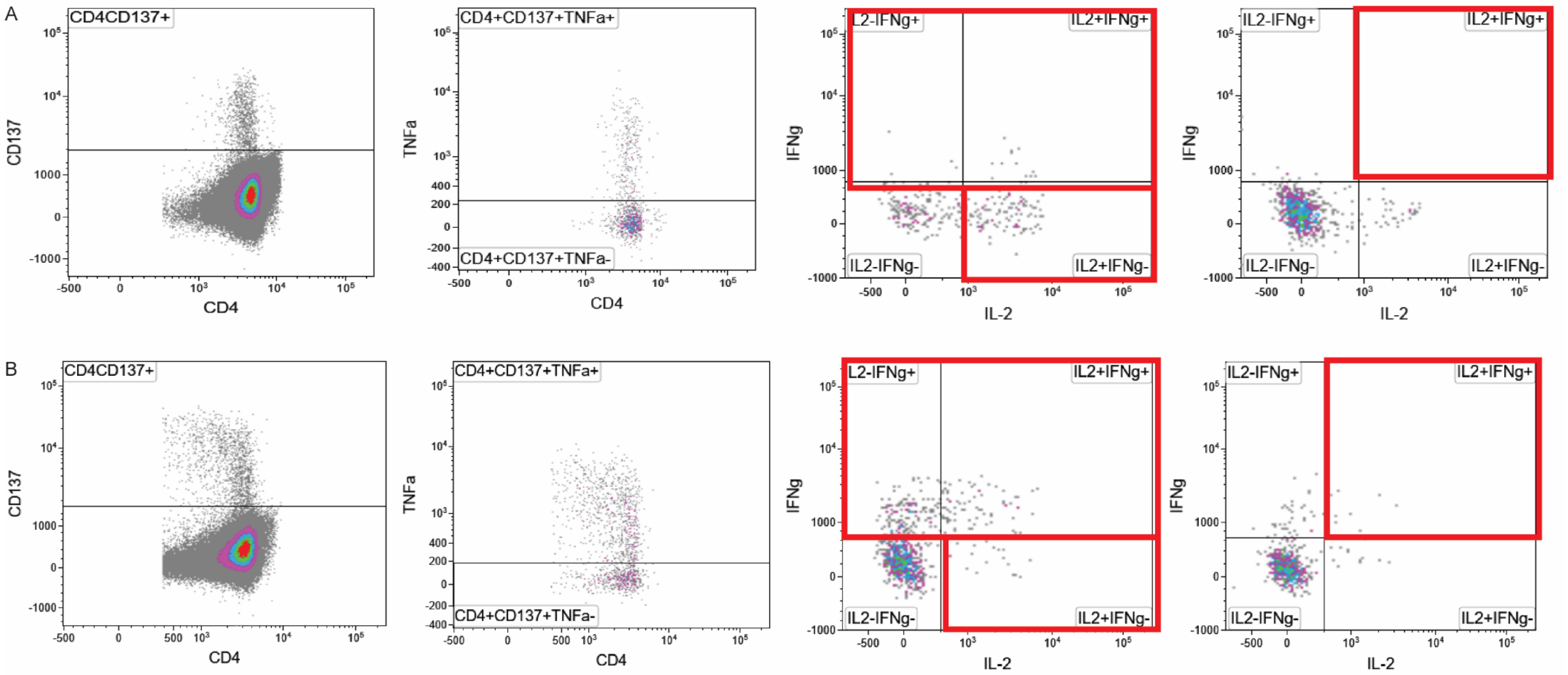

Supplementary Figure 2 Analysis of polyfunctional antigen-reactive T cells

A typical example of the gating strategy applied to analyze proportions of polyfunctional (i.e. producing 2 or more pro-inflammatory cytokines) antigen-reactive (CD137-expressing CD4+T cells is given. In the top panel (A), alloreactive CD4+ T cells are identified using CD137 (first plot) and these are subsequently dissected into those producing TNFα and those not in the second plot. in the third plot, those producing TNFα were subdivided into 4 quadrants by plotting IL2 (X-axis) against IFNγ (Y-axis) whereas those lacking TNFα are subdivided into 4 quadrants using IL2 on X-axis and IFNγ on Y-axis in the fourth plot. The red squares highlight the cells producing multiple cytokines.
In the panel at the bottom (B) the same approach was chosen to characterize virus-reactive-expressing CD4+ T cells.
Moreover, this approach was also applied for evaluating proportions of polyfunctional antigen-reactive CD8+ T cells.

A CD25++CD127- Treg staining within CD4+ T cells prior to stimulation (day 0)

B CD25++CD127- Tregs within CD4+ T cells upon 18-24 hours stimulation (day 1)

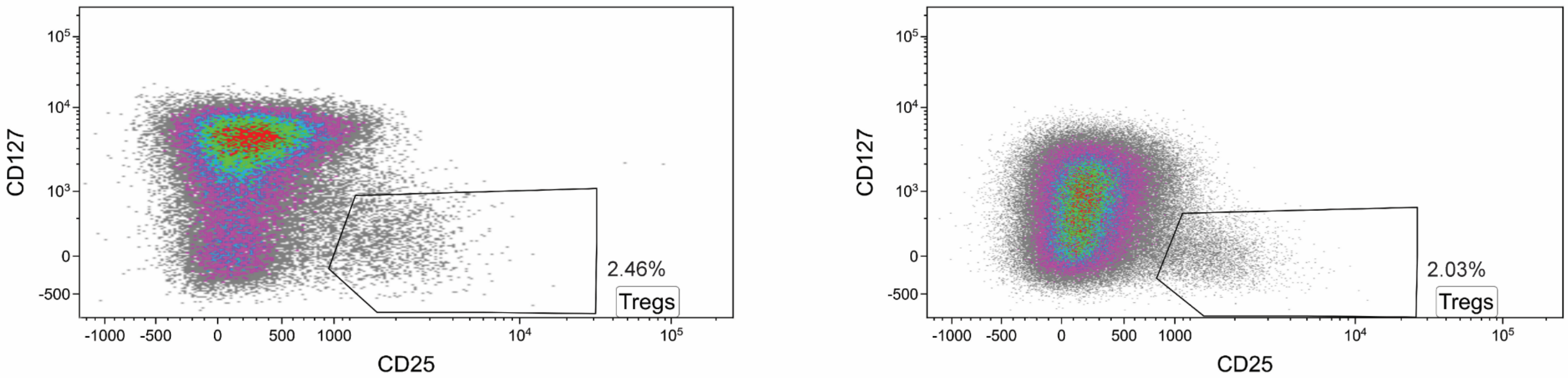

Supplementary Figure 3 Representative example of identification of regulatory T cells

PBMCs of kidney transplant recipients were stained prior to stimulation with antibodies directed against CD25 (BV711-labelled anti CD25, clone M-A251) and CD127 (PE-Cy7-labelled anti CD127, clone A019D5) to allow for identification of regulatory CD4+ T cells following short-term stimulation with donor-antigen.
In **A**, CD4+ Tregs at day 0 are depicted whereas in **B**, the identification of these Tregs, without restaining, are given following stimulation with donor-antigen.

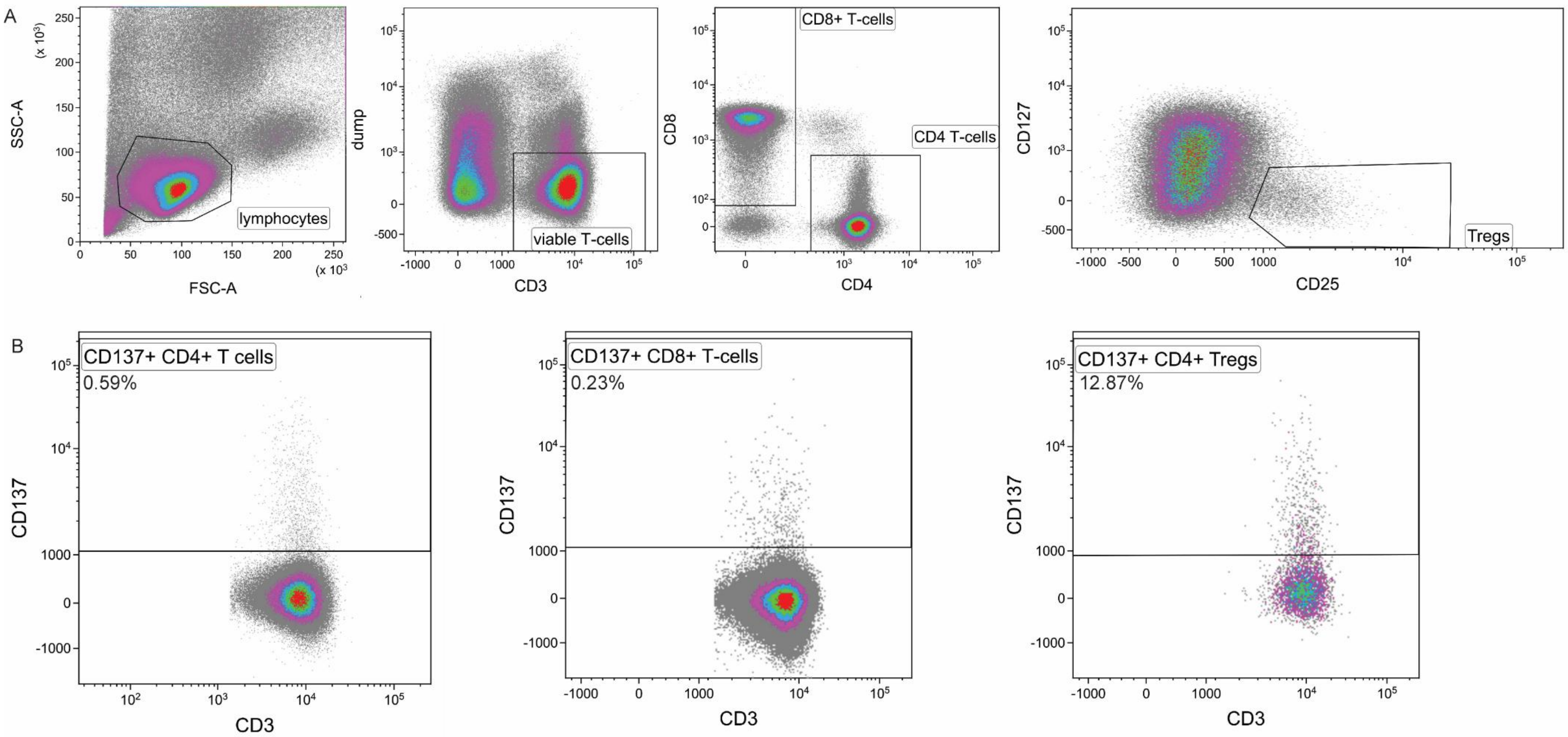

Supplementary Figure 4 Analysis of alloreactive T cells

Representative example of the gating strategy applied for analysis of proportions of alloreactive T cell subsets is depicted.
In the top panel (**A**), lymphocytes are selected (first plot) based on forward-sideward characteristics. These lymphocytes are than plotted for CD3 (X-axis) and using a dumpchannel, consisting of antibodies directed against, CD14, CD19, CD56 and the live-dead stain FVS780, viable T cells are selected (second plot). In the third plot, CD4 and CD8+ T cells are identified whereas within the fourth plot, regulatory CD4+ T cells are identified using CD25 (X-axis) and CD127 (Y-axis). The bottom panel (**B**), illustrates identification of donor-reactive CD4+ (left plot), CD8+ (middle plot) T-cells and CD4+Tregs (right plot) using expression of CD137 within the different T cell subsets.

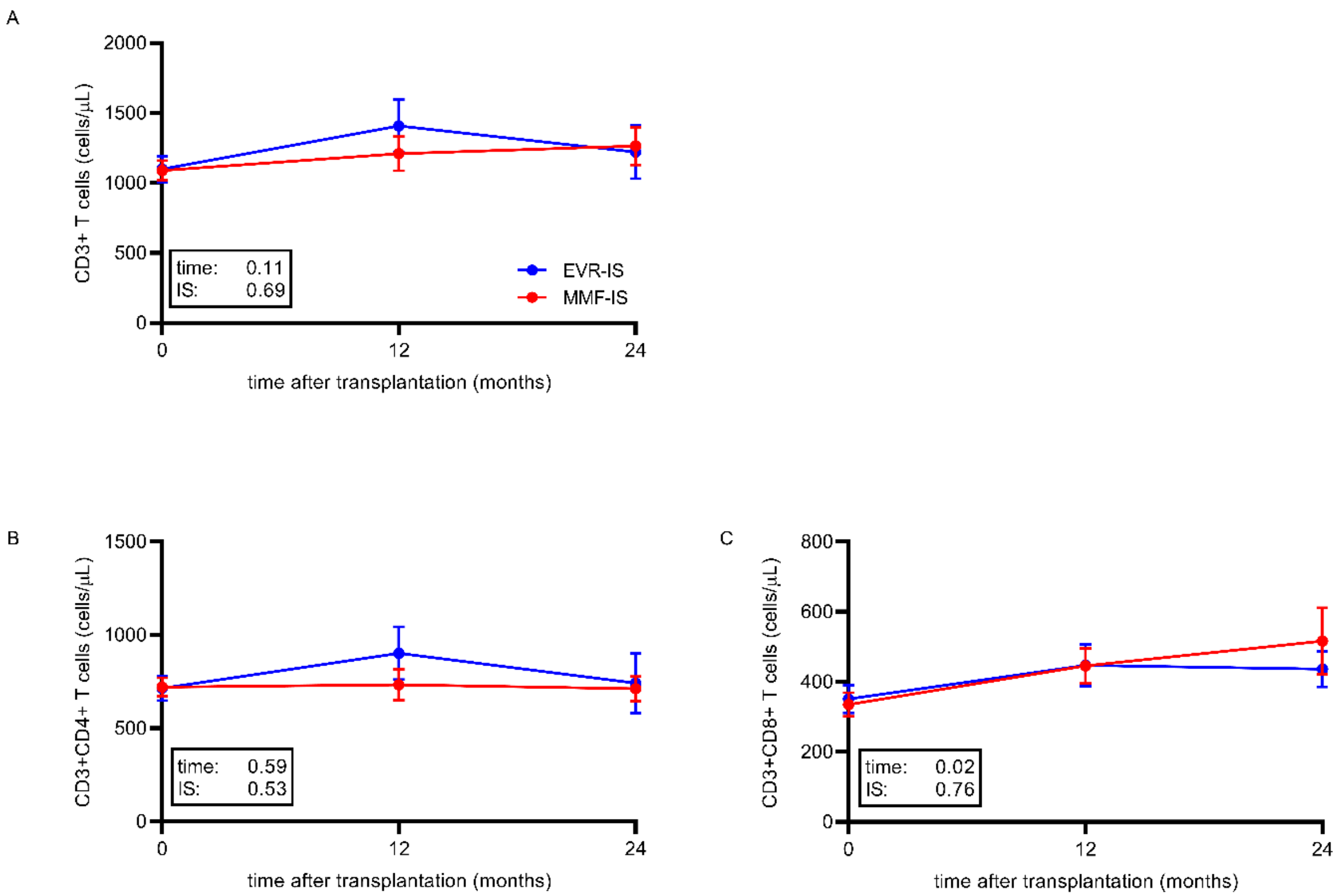

Supplementary Figure 5 Absolute number of T cell subsets

Absolute number of CD3+, CD4+ and CD8+ T cells were determined by flowcytometry using BD Trucount™ tubes, containing a fixed number of beads and 50 μL whole blood of 39 kidney transplant recipients on EVR-IS (blue lines) and 41 recipients on MMF-IS (red lines). On Y-axis absolute numbers of CD3+ (A), CD4+ (B) and CD8+ (C) T cells are depicted in cells/μL. On the X-axis, the different timepoints after transplantation are depicted in months. Values depicted are mean ± SEM. Mixed effect analysis with Tukey multiple comparison test was used to determine effect of time and treatment arm (IS) on the kinetics of T cell subsets (P-values are listed in square) and to compare the difference between both treatment arms for each time-point.

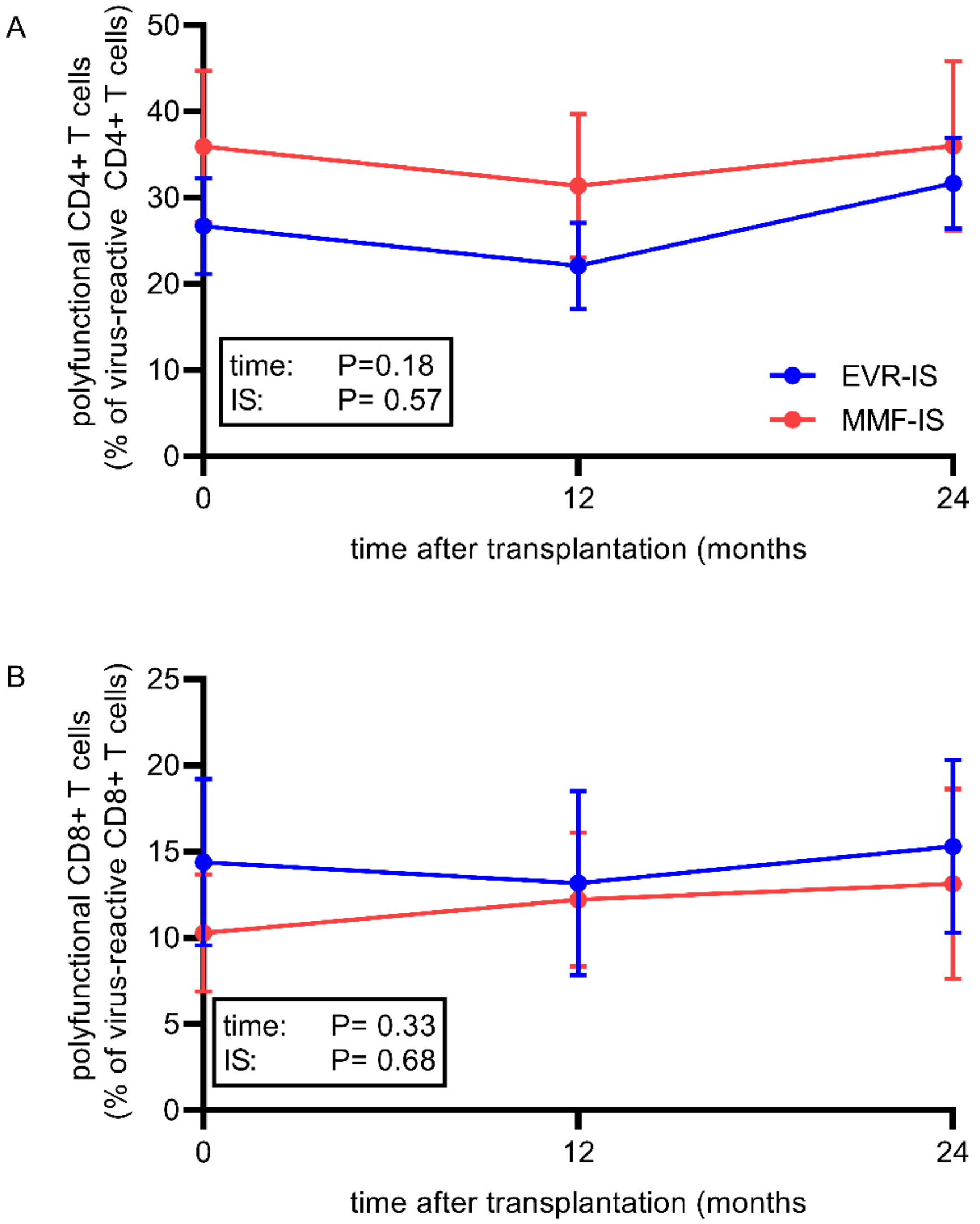

Supplementary Figure 6 Proportions of polyfunctional virus-reactive T cells
Proportions of polyfunctional virus-reactive CD4+ (A) and CD8+ (B) T cells are depicted for kidney transplant recipients (N=25) prior to and at M12 and M24 after transplantation. Data are depicted as mean± SEM and blue lines depict kidney transplant recipients on EVR-IS (N=12) whereas the red lines represent those on MMF-IS (N=13). Mixed effect analysis was used with Tukey multiple comparison test to determine effect of time and treatment arm on the kinetics of T cell subsets (P-values are listed in square) and to compare the difference between both treatment arms for each time-point.
